# Adult Stem Cells Properties in Terms of Commitment, Aging and Biological Safety of Grit-Blasted and Acid-Etched Ti Dental Implants Surfaces

**Published:** 2014

**Authors:** Chiara Gardin, Letizia Ferroni, Eriberto Bressan, José L. Calvo - Guirado, Marco Degidi, Adriano Piattelli, Barbara Zavan

**Affiliations:** 1 *Department of Biomedical Sciences, University of Padua, Padua, Italy.*; 2 *Department of Neurosciences, University of Padua, Padua, Italy.*; 3 *Department of General Dentistry, Faculty of Medicine and Dentistry, University of Murcia, Murcia, Spain.*; 4 *Private Practice, Bologna, Italy.*; 5 *Department of Medical, Oral and Biotechnological Sciences, University of Chieti, Italy.*

**Keywords:** Titanium dental implants, surface properties, adipose- derived stem cells, biocompatibility, osteogenic differentiation

## Abstract

Titanium (Ti) is one of the most widely used biomaterials for manufacturing dental implants. The implant surface properties strongly influence osseointegration. The aim of the present study was to *in vitro* investigate the characteristics of Ti dental implants in terms of mutagenicity, hemocompatibility, biocompatibility, osteoinductivity and biological safety. The Ames test was used to test the mutagenicity of the Ti dental implants, and the hemolysis assay for evaluating their hemocompatibility. Human adipose - derived stem cells (ADSCs) were then seeded onto these implants in order to evaluate their cytotoxicity. Gene expression analyzing with real-time PCR was carried out to investigate the osteoinductivity of the biomaterials. Finally, the genetic stability of the cells cultured onto dental implants was determined by karyotyping. Our results demonstrated that Ti dental implants are not mutagenic, do not cause hemolysis, and are biocompatible. The MTT assay revealed that ADSCs, seeded on Ti dental implants, proliferate up to 30 days in culture. Moreover, ADSCs loaded on Ti dental implants show a substantial expression of some osteoblast specific markers, such as COL1A1, OPN, ALPL, and RUNX2, as well as chromosomal stability after 30 days of culture in a medium without osteogenic factors. In conclusion, the grit-blasted and acid-etched treatment seems to favor the adhesion and proliferation of ADSCs and improve the osteoinductivity of Ti dental implant surfaces.

Titanium (Ti) is one of the most widely used biomaterials for dental implants (1, 2) because of its excellent mechanical strength and chemical stability (3). In addition, the low-toxicity and the low rate of ion release from its surface make Ti a highly biocompatible material (4, 5).

The clinical success of Ti dental implants is their osseointegration, which is the formation of a strong connection between the implant surface and the surrounding host bone (6, 7). It is now well documented that the surface properties of Ti implants, such as wettability, charge, chemistry and topography, are the most influencing factors in the establishment of cell-biomaterial contacts and in the improvement of osseointegration (8-11). In particular, cell attachment, proliferation and differentiation into an osteoblastic phenotype seem to be strongly regulated by the surface roughness of dental implants (12-14). Plasma- spray coatings, grit- blasting, acid- etching, electrochemical processes or a combination of them are the most frequently used techniques to obtain Ti rough surfaces (15, 16). Grit- blasting is usually achieved by treating the implant surface with hard ceramic, such as alumina, titanium oxide and calcium phosphate particles (17-19). Various sizes of these ceramic particles generate different roughness on Ti implants surfaces. Another method for obtaining rough surfaces consists in treating Ti dental implants with strong acids, such as HCl, H_2_SO_4_, HNO_3_ and HF (20). This chemical process, known as acid- etching, improves the osteoconductive properties of implants enhancing osteoblasts adhesion, thus resulting in bone formation directly on the surface of the implant (21). However, the effects of acid- etching on the long- term stability of the Ti dental implant are rather limited. Indeed, the acid- etching technique causes hydrogen embrittlement, which leads to microcracks on the surface of the titanium dental implant. Such cracks compromise the good mechanical properties, especially fatigue resistance, of the Ti implant (22). To avoid this drawback, acid- etching is used in combination with grit- blasting: the result is an implant surface both macrotopographically wavy and rough at the microlevel (23). *In vitro* and *in vivo* studies demonstrated that grit-blasted and acid- etched surfaces show great biomechanical stability, high mechanical resistance, low risk of clinical failures, and high bond between implant and bone (24, 25).

Although research is investing significantly on developing new Ti modified surfaces, a detailed understanding of the molecular and cellular mechanisms of osseointegration is still lacking. Traditionally, bone regeneration around Ti dental implants is considered a process comparable to healing after a fracture (26). The healing process always occurs through a series of three overlapping events: inflammation, proliferation, and remodeling (27). In all these events, an important role is carried out by mesenchymal stem cells (MSCs), which have self- renewal capacity and multi-lineage potential. For example, MSCs are able to differentiate into osteoblasts, which are the cells responsible of bone growth (28). In the presence of an implant, it is crucial that these cells adhere to the dental implant surface in order to develop a bone- specific extracellular matrix (ECM), which later mineralizes to form an integrated bone- implant interface (23).

The aim of this study was to investigate the influence of the grit- blasted and acid- etched Ti implants surface on the biological response of human MSCs derived from adipose tissue (ADSCs) by means of *in vitro* tests. Initially, the mutagenicity and the hemocompatibility of Ti dental implants were investigated. Then, their cytotoxicity towards human ADSCs, as well as the chromosomal stability of the cells seeded onto these surfaces, were evaluated.

## Material and Methods

**Biomaterials**

In this study, Ti dental implants crew shaped and with grit- blasted and acid- etched surfaces (3- 4 mm diameter and 11 mm length; XiVE^®^ S plus Screw Implant, Friadent^®^, Dentsply, Mannheim, Germany) were used. All dental implants used were sterilized by γ- rays.

**Ames test**

The mutagenic potential of Ti implants was evaluated by the Ames test performed with the Salmonella mutagenicity complete test kit (Moltox, Molecular toxicology Inc., Boone, NC, USA). Nutrient Broth (blank) was used as the extraction vehicle; aluminium oxide ceramic rod (VITA In- Ceram Alumina CA-12, CE 0124, lot 15320) was used as negative control; ICR 191 acridine (Moltox, 60- 101) and sodium azide (Moltox, 60- 103) were used as positive controls. Extraction conditions were (24± 2 h at 37± 1°C). Three replicates were performed for each sample. The bacteria plates were incubated with the different extracts for 48 h at 37°C, then the number of revertant colonies per plate was counted. Interpretation of results was as follows: negative (not mutagenic) if the number of reverted colonies was equivalent to those observed with blank and negative controls; positive (mutagenic) if the number of reverted colonies was equivalent to those observed with positive controls.

**Hemolysis assay**

The blood compatibility of Ti implants was evaluated by the hemolysis assay performed following standard practices set forth in ASTM F756. Blood was obtained from three healthy New Zealand rabbits, pooled, then diluted in PBS to a total hemoglobin concentration of 10± 1 mg/ ml. One ml of diluted rabbit blood was added to 7 ml of the following PBS extracts. For the extraction of the test material, triplicate 2 gr portions of Ti implants were covered with 10 ml PBS. For the negative control, triplicate 30 cm^2^ portions of high density polyethylene (HDPE) were covered with 10 ml of PBS. For the positive control, triplicate 10 ml portions of sterile water for injection (SWFI) were used. Extraction conditions were 50 °C for 72 h for all samples. Each tube was incubated for 3 h at 37 °C with periodic inversions. Following incubation, the tubes were centrifuged for 15 min at 800 g. A 1 ml aliquot of the resulting supernatant from test materials, negative and positive controls was added to 1 ml of Drabkin’s reagent (Sigma- Aldrich) and incubated at room temperature for 15 min. The reaction product between hemoglobin and Drabkin’s reagent is a cyanoderivative that was quantified by measuring absorbance at 540 nm with a multilabel plate reader (Victor 3 Perkin Elmer, Milano, Italy). The hemolysis index (HI) was then calculated using the mean absorbance value (OD) for each group as follows:

HI (%) = OD (test material)- OD (negative control) / OD (positive control)- OD (negative control)× 100.

The implant was considered as non- hemolytic if the HI was 2% or less.

**Human stem cells isolation**

Human adipose- derived stem cells (ADSCs) were isolated from the adipose tissue of healthy patients (age: 21-36 years; BMI: 30-38) undergoing cosmetic surgery procedures according to the guidelines of the plastic surgery clinic at the University of Padova. Written informed consent was obtained from all patients, in accordance with the Helsinki Declaration, before their inclusion in this study. The Ethical Committee of Padua Hospital approved the research protocol.

The adipose tissues were digested and the cells isolated, expanded and seeded as previously described (29). Briefly, the adipose tissue was washed with phosphate buffered saline (PBS, EuroClone, Milan, Italy) and digested using a solution of 0.075% collagenase from Clostridium histolyticum type II (Sigma- Aldrich, St. Louis, MO, USA) in Hank's balanced salt solution (HBSS, Lonza S.r.l., Milano, Italy), for 3 h at room temperature and under slow agitation. At the end of the digestion, the collagenase activity was blocked with an equal volume of cDMEM which consisted of Dulbecco’s modified Eagle’s medium (DMEM, Lonza S.r.l.) supplemented with 10% fetal bovine serum (FBS, Bidachem S.p.A., Milano, Italy) and 1% Penicillin/ Streptomycin (P/S, EuroClone). After centrifugation for 4 min at 1200 rpm, the pellet was washed in PBS and filtered with a 70 µM cell strainer (BD Biosciences, Mississauga, Ontario, Canada). The cell suspension was resuspended in cDMEM, transferred to a 25 cm^2^ tissue culture flask, then incubated at 37 °C and 5% CO2. After 3 days, floating cells were discarded and fresh medium was added on the adherent cells. At confluence, ADSCs were harvested by trypsin treatment, then cultivated up to passage 3 (p3). At this point, flow cytometry analyzes were performed for evaluating the stemness of these cells: ADSCs resulted positive for CD 73, CD 90 and CD 105 antibodies; negative for CD 34 antibody (data not shown).

**Cells seeding onto Ti implants**

ADSCs at p4 were seeded onto the Ti implants at a density of 2x 10^6^ cells/ implant in a 12- well plate. The cells were cultured in cDMEM without any osteogenic differentiation factor at 37 °C with 5% CO_2_ up to 30 days, and the medium was changed twice a week.

At the same time, 1x 10^4^ cells were seeded on a polystyrene 24- well plate in the presence of cDMEM or osteogenic differentiation medium (EuroClone) and cultured for 15 days. These cells were used as control for normalization of gene expression data.

**MTT assay**

To determine the proliferation rate of cells grown on Ti implants, the MTT- based (methyl thiazolyl- tetrazolium) cytotoxicity assay was performed according to the method of Denizot and Lang with minor modifications (30). The test is based on mitochondria viability, i.e., only functional mitochondria can oxidize an MTT solution, giving a typical blue- violet end product. After harvesting the culture medium, the cells were incubated for 3 h at 37 °C in 1 mL of 0.5 mg/ mL MTT solution prepared in PBS solution. After removal of the MTT solution by pipette, 0.5 mL of 10% dimethyl sulfoxide in isopropanol (iDMSO) was added for 30 min at 37 °C. For each sample, absorbance values at 570 nm were recorded in duplicate on 200 μL aliquots deposited in 96- well plates using a multilabel plate reader (Victor 3 Perkin Elmer). All samples were examined after 15 and 30 days of culture.

**RNA extraction and first strand cDNA synthesis**

Total RNA was extracted with RNeasy Mini Kit (Qiagen GmbH, Hilden, Germany), including DNase digestion with the RNase- free DNase set (Qiagen), from ADSCs seeded onto Ti implants for 15 and 30 days. The RNA quality and concentration of the samples were measured using the NanoDropTM ND-1000 (Thermo Scientific).

For the first strand cDNA synthesis, 200 ng of total RNA of each sample was reverse transcribed with M-MLV Reverse Transcriptase (Invitrogen, Carlsbad, CA, USA), following the manufacturer’s protocol.

**Real- time PCR**

Human primers were selected for each target gene with Primer 3 software (Table 1). Real-time PCRs were carried out using the designed primers at a concentration of 300 nM and FastStart SYBR Green Master (Roche Diagnostics, Mannheim, Germany) on a Rotor- Gene 3000 (Corbett Research, Sydney, Australia). Thermal cycling conditions were as follows: 15 min denaturation at 95 °C; followed by 40 cycles of denaturation for 15 sec at 95 °C; annealing for 30 sec at 60 °C; and elongation for 20 sec at 72 °C. Differences in gene expression were evaluated by the 2∆∆Ct method (31) using ADSCs cultured in cDMEM onto tissue culture polystyrene as control. The expression level of the selected genes were also evaluated for ADSCs seeded onto tissue culture polystyrene in the presence of osteogenic differentiation medium (EuroClone). Values were normalized to the expression of the glyceraldehyde- 3-phosphate dehydrogenase (GAPDH) internal reference, whose abundance did not change under our experimental conditions.

**Karyotype analysis**

**Table 1:** Human primers sequences

*gene symbol*	*forward primer (5’→ 3’)*	*reverse primer (5’→ 3’)*	*product length (bp)*
ALPL	GGCTTCTTCTTGCTGGTGGA	CAAATGTGAAGACGTGGGAATGG	181
COL1A1	TGAGCCAGCAGATCGAGA	ACCAGTCTCCATGTTGCAGA	178
GAPDH	TCAACAGCGACACCCAC	GGGTCTCTCTCTTCCTCTTGTG	203
OCN	GCAGCGAGGTAGTGAAGAGAC	AGCAGAGCGACACCCTA	193
ON	TGCATGTGTCTTAGTCTTAGTCACC	GCTAACTTAGTGCTTACAGGAACCA	183
OPN	TGGAAAGCGAGGAGTTGAATGG	GCTCATTGCTCTCATCATTGGC	192
PPARG	CAGGAGATCACAGAGTATGCCAA	TCCCTTGTCATGAAGCCTTGG	173
RUNX2	AGCCTTACCAAACAACACAACAG	CCATATGTCCTCTCAGCTCAGC	175

ALPL, alkaline phosphatase, liver/bone/kidney, COL1A1, collagen, type I, alpha 1, GAPDH, glyceraldehyde-3-phosphate dehydrogenase, OCN, osteocalcin, ON, osteonectin, OPN, osteopontin , PPARG, peroxisome proliferator-activated receptor gamma, RUNX2, runt- related transcription factor 2

**Table 2:** Mutagenicity evaluation by the Ames test

	**STDisc™ TA1535**		**STDisc™ TA1537**			**STDisc™ TA98**		**STDisc™ TA100**		
***sample***	***rev/plate*** a	***result***	***rev/plate*** a		***result***	***rev/plate*** a	***result***	***rev/plate*** a		***result***
blank	4 ± 3	not mutagenic		5 ± 3	not mutagenic	5 ± 3	not mutagenic		3 ± 3	not mutagenic
NC	3 ± 2	not mutagenic		4 ± 2	not mutagenic	2 ± 2	not mutagenic		4 ± 2	not mutagenic
PC1	922 ± 76	mutagenic		928 ± 76	mutagenic	921 ± 76	mutagenic		929 ± 76	mutagenic
PC2	847 ± 50	mutagenic		851 ± 50	mutagenic	844 ± 50	mutagenic		849 ± 50	mutagenic
TS	3 ± 2	not mutagenic		4 ± 2	not mutagenic	2 ± 2	not mutagenic		4 ± 2	not mutagenic

a Number of revertants/plate: mean of three independent experiments ± SD, NC, negative control: aluminium oxide ceramic rod, PC1, positive control 1: ICR 191 Acridine, PC2, positive control 2: Sodium Azide, TS, tested sample: Ti implant

After 30 days of culture on Ti implants, cells were exposed to colchicine (Sigma-Aldrich, St. Louis, MO, USA) for 6 h, washed in PBS, dissociated with trypsin (Lonza S.r.l), and centrifuged at 300 g for 5 min. The pellet was carefully resuspended and incubated in 1% sodium citrate for 15 min at 37 °C, then fixed and spread onto -20 °C cold glass slides. Metaphases of cells were Q-banded and karyotyped in accordance with the international system for human cytogenetic nomenclature recommendations. Twenty five meta-

phases were analyzed for three expansions.

**Statistical analyzes**

One- way analysis of variance (ANOVA) was used to analyze the data. Repeated measures ANOVA with a post- hoc analysis using Bonferroni’s correction for multiple comparisons was performed, and t-tests were used to determine signifycant differences P<0.05). Repeatability was calculated as the stan-dard deviation of the difference between measurements. All testings were performed using SPSS 16.0 software (SPSS Inc, Chicago, Illinois, USA) (licensed by the university of Padova). 

**Table 3:** Blood compatibility evaluation by the hemolysis assay

***sample***	***OD*** ^a^	***HI*** ^b^	***result***
PC1	0.8762 ± 0.012	100%	hemolytic
NC	0.0143 ± 0.002	0%	nonhemolytic
TS	0.0144 ± 0.002	0,046%	nonhemolytic

OD, absorbance value at 540 nm: mean of three independent experiments ± SD, HI, hemolysis index***,*** PC, positive control: Sterile Water for Injection (SWFI), NC, negative control: High Density PolyEthylene (HDPE), TS, tested sample: Ti implant

**Fig. 1:**
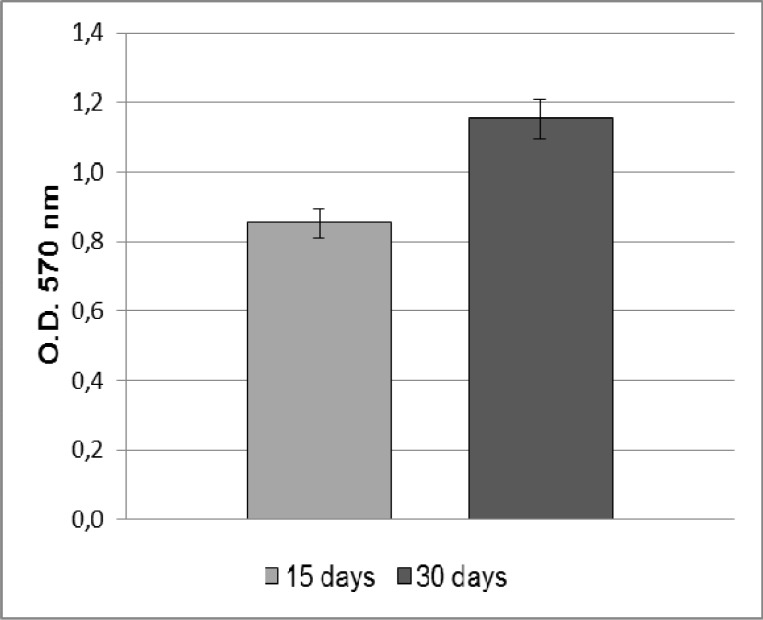
MTT assay of ADSCs cultured on the Ti dental implants. ADSCs proliferation rate increase during the culturing time, reaching the maximum value at 30 days

**Fig. 2:**
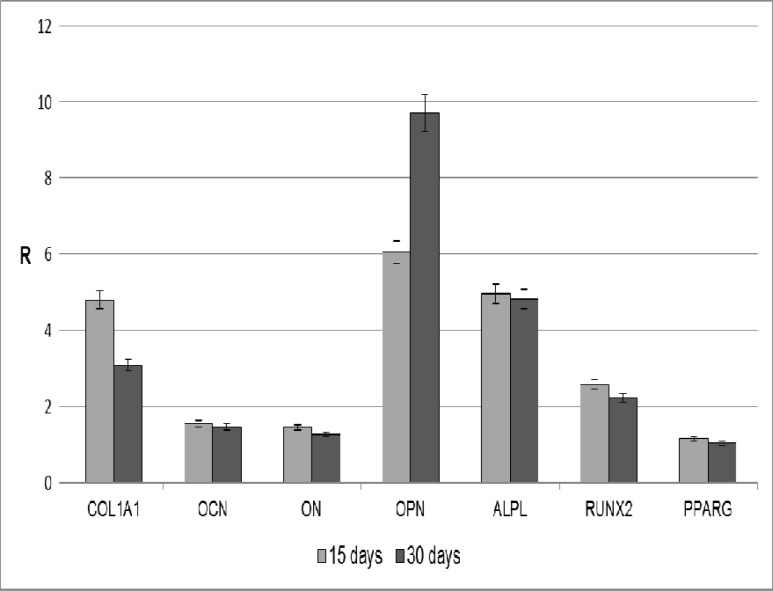
Osteoblast markers expression in ADSCs cultured in cDMEM on the Ti implants. The results are reported as ratios (R) with respect to the mRNA expression of ADSCs seeded in tissue culture on polystyrene for 15 days in cDMEM

## Results

**Evaluation of the mutagenicity of Ti dental implants**

The Ames test was performed in order to assess the mutagenic potential of Ti implants. Four different histidine dependent mutant strains (TA1535, TA1537, TA98 and TA100) of *Salmonella typhimurium* were used. As reported in table 2, no mutagenic activity has been revealed.

**Evaluation of the hemocompatibility of Ti discs**

The hemolysis assay was performed in order to evaluate the blood compatibility of the Ti implants, which are intended for blood contacting applications. The HI was less than 2%, indicating the absence of any hemolytic activity of the tested material (Table 3).

**Biocompatibility of Ti implants**

In order to evaluate the biocompatibility of Ti implants, ADSCs were seeded and cultivated onto these surfaces up to 30 days. The results of MTT assay show that the cells were able to adhere and proliferate onto the Ti implants (Fig. 1).

**Expression of osteoblast markers**

The gene expression level of some osteoblast markers were analyzed at day 15 and 30 by means of real- time PCR in order to verify the osteoinductive properties of the Ti implants used in the present study. The expression of selected genes (ALPL, COL1A1, OCN, ON, OPN, RUNX2, and PPARG) were evaluated in relation to the expression of a reference gene (GAPDH). Cells seeded on tissue culture polystyrene in cDMEM for 15 days were used as control for data normalization. As shown in figure 2, the expression of some osteoblast markers in ADSCs seeded onto the Ti dental implants is higher compared to the control condition. In particular, high gene expression levels were observed for COL1A1, OPN, ALPL, and RUNX2.

**Fig. 3:**
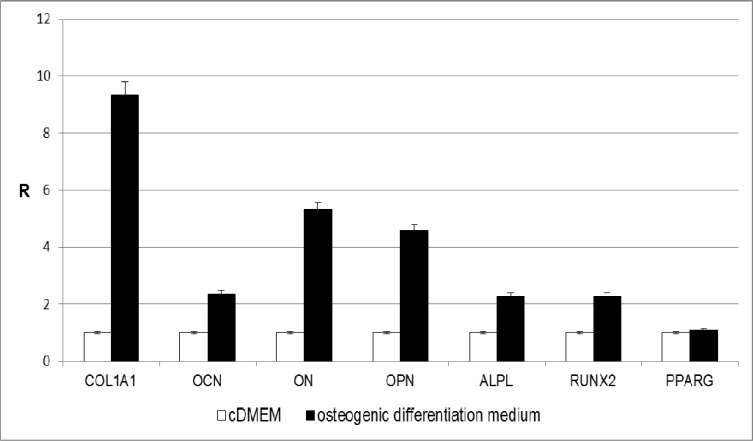
Effect of osteogenic differentiation medium on osteoblast markers expression in 15 days cultured ADSCs. The results are reported as ratios (R) with respect to the mRNA expression of ADSCs seeded in tissue culture polystyrene for 15 days in the presence of cDMEM

Similar results were obtained when comparing the expression level of the same markers in ADSCs seeded on tissue culture plates in the presence of osteogenic differentiation medium to the control (Fig.3). Also in this case, the expression of COL1A1, OPN, ALPL, and RUNX2 was higher in cells cultivated with osteogenic factors as opposed to the control condition (cDMEM). In addition, in ADSCs treated with the osteogenic medium an increase in OCN and ON mRNA expression was also detected.

**Cytogenetic analysis**

**Fig. 4:**
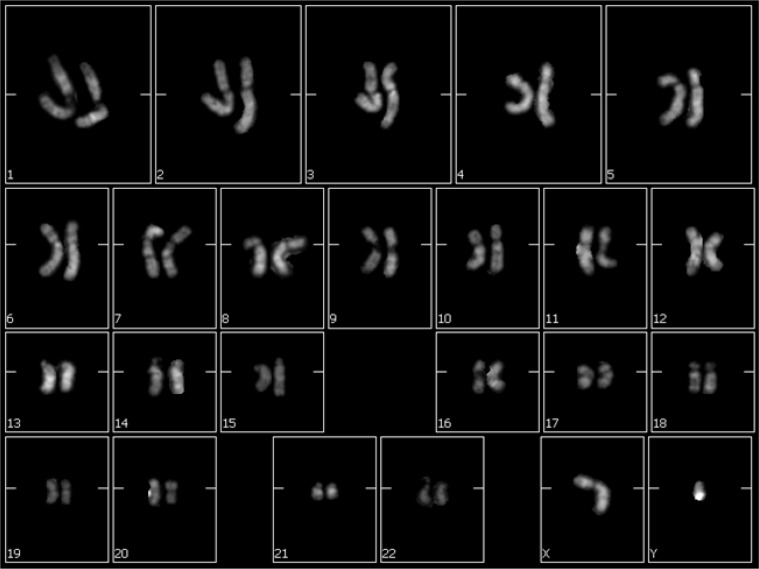
Karyotype analysis of ADSCs seeded on the Ti implants for 30 days. No chromosomal alterations are present

The chromosomal stability of ADSCs seeded on the Ti implants was analyzed by means of karyotyping. As reported in figure 4, no chromosomal alterations are present in ADSCs seeded onto these surfaces for 30 days.

## Discussion

Ti and its alloys are the most commonly used biomaterials in dental implantology. Nevertheless, a question that remains to be answered is how molecular and cellular events are influenced by the material surface properties. In this study, we have analyzed the effects of Ti dental implants with grit- blasted and acid- etched surfaces on the behavior of MSCs isolated from human adipose tissue (ADSCs). Preliminary analyses were performed to test the mutagenicity and hemocompatibility of the Ti dental implants. Subsequently, ADSCs were seeded onto these surfaces to evaluate their biocompatibility and osteoinductive properties. Finally, the safety of the biomaterials was investigated by means of karyotyping.

The first experiments were carried out to assess whether the treatments of Ti implants have mutagenic potential. There is considerable evidence that gene mutations are involved in cancer formation in humans. The mutagenic potential of Ti implants was examined with the Ames test (32). In the present study, four Salmonella typhimurium strains were used: TA1535 and TA100, which result from a base-pair substitution; TA1537 and TA98, products of a frameshift mutation. In this way, it was possible to identify mutagens acting with different mechanisms. The four Salmonella strains were incubated with extracts deriving from Ti implants for 48 h. The mutagenicity of a substance is proportional to the number of colonies observed. The low number of histidine revertant colonies indicates that Ti implants lack mutagenic activity at the conditions tested.

At this point, we performed the hemolysis assay which is considered to be a very simple and reliable test for estimating blood compatibility of materials. The test relies on the measurement of free hemoglobin released into the plasma when blood cells are damaged. Generally, the smaller the HI, the better the blood compatibility of the biomaterial. The material extract tested in this study induced less than 2% of contacting erythrocytes to hemolyze over 3 h of contact with blood. These results indicate that Ti implants have no hemolytic effects and meet the requirements for clinical application.

In the process of bone healing and implant osseointegration, MSCs are the key repair cells, and their cellular response is important because successful osseointegration of implants depends on the adhesion of MSCs onto the implant surface (33). In this study, human ADSCs were used to evaluate the cytotoxicity of Ti implants. The results of the MTT assay indicate that ADSCs are able to attach and grow on Ti implants and that cell proliferation rate increases during the culturing time, reaching the maximum value after 30 days. It seems that grit- blasted and acid- etched treatment of Ti surfaces positively affects cell proliferation.

As explained before, the regeneration of bone is regulated by a series of complex events that involve the sequential cascade of ECM proteins production and its subsequent controlled calcification (34). These proteins include collagens as well as non- collagenous proteins (35). In order to evaluate the osteoinductivity of Ti implants on osteoblast differentiation of ADSCs, the expression of osteogenic specific markers were evaluated with real- time PCR. Collagen type I (COL1A1) represent 90% of the total bone protein content (36). When ADSCs are cultured on the Ti implants, the gene expression of COL1A1 is found to be significantly up- regulated. Such a result is very interesting since COL1A1 synthesis is known to be a prerequisite for ECM formation and minerali-zation in bone (37).

Osteocalcin (OCN), osteonectin (ON) and osteopontin (OPN) are the non- collagenous proteins of bone, which collectively contribute to the bone mineralization. OCN, a specific osteoblast protein, is the most abundant non- collagenous protein found in bone ECM after collagens. It is thought that OCN is implicated in bone mineralization and calcium ion homeostasis (38). ON is a glycoprotein that binds calcium (39). It is secreted by osteoblasts during bone formation, initiating mineralization and promoting mineral crystal deposition. ON also shows affinity for collagen in addition to bone mineral calcium. In this study, the expression levels of both OCN and ON are similar at 15 and 30 days of culture.

Although no significant changes are found in the expression of OCN and ON, other markers associated with the osteogenic differentiation are up-regulated. For example, the gene expression of OPN and alkaline phosphatase (ALPL) is strongly increased in ADSCs cultured onto Ti implants both at 15 and 30 days. OPN is an important factor in bone remodeling (40), and different studies have shown that it plays a role in anchoring osteoclasts to the mineral matrix of bones (41). Alkaline phosphatase (ALPL) is a membrane- bound protein with the catalytic domain on the osteoblastic plasmalemma. It is a marker of early osteogenic development and has probably an initiator and regulator role in calcification (42). The elevated OPN and ALPL expression observed in this study supports the success of the osteoblastic differentiation of ADSCs and may be an indication of the osteoinductive properties of the scaffolds used.

The expression of transcription factor genes are essential for cellular commitment to a specific differentiation lineage (43-45). Many studies have confirmed the existence of an inverse reciprocal relationship between adipogenesis and osteogenesis (46-49). Osteoblast differentiation requires expression of the osteoblast- specific transcription factor runt- related transcription factor 2 (RUNX2) (50-52). Likewise, adipogenic differentiation is regulated by peroxisome proliferator- activated receptor gamma (PPARG), which also possesses anti-osteoblastogenic effects (53, 54). In this study, ADSCs seeded onto Ti implants showed high expression level of RUNX2 both at 15 and 30 days. On the contrary, PPARG expression did not change over time. Such a result might indicate that Ti dental implants are able to stimulate the differentiation of ADSCs towards the osteogenic phenotype while suppressing the adipogenic commitment of these cells. This is in line with the hypothesis that increased expression of one transcription factor is typically associated with down- regulation of the other (47-49).

At the same time, high mRNA expression of osteogenic markers were obtained when ADSCs were cultured on tissue culture plates in the presence of a differentiation medium supplemented with osteogenic factors. Indeed, the gene expression level of COL1A1, OCN, ON, OPN, ALPL and RUNX2 was significantly higher compared to the control condition, that is ADSCs seeded in monolayer with cDMEM. On the contrary, the expression of PPARG did not change under these culture conditions.

Taken together, our results demonstrate that the osteogenic differentiation of ADSCs may be dependent on the Ti implant surface characteristics, which have effects similar to the addition of osteogenic growth factors in monolayer ADSCs cultures.

In order to evaluate the chromosomal stability of ADSCs maintained in culture 30 days on the Ti implants, we performed karyotyping. This method consisted in the analysis of metaphases of cells for testing the presence of chromosomes alterations following their proliferation and differentiation onto the Ti implants. No chromosomal alterations were found in the karyotype of ADSCs seeded on Ti implants for 30 days. This confirms that the cells are able to maintain their chromosomal stability, an extremely important fact when considering possible clinical use (55).

In conclusion, our results indicate that Ti implants are not mutagenic and do not cause hemolysis. Moreover, their surfaces are found to be biocompatible and not toxic when seeded with human ADSCs. Rather, the grit- blasted and acid- etched treatment seem to favor the adhesion and proliferation of these cells. The osteoinductivity of Ti implants has been determined by the osteogenic commitment of ADSCs in absence of a differen-tiation medium. Finally, the maintenance of chro-mosomal stability by ADSCs seeded on the Ti implants ensures the biological safety of these materials.
